# Concomitant uveal melanoma and papillary thyroid carcinoma: a case report

**DOI:** 10.1186/s13256-022-03258-1

**Published:** 2022-01-18

**Authors:** Seiiedeh Samaneh Taghian Jamaleddin Kolaii, Amir Reza Dehghanian, Marjan Jeddi

**Affiliations:** 1 Endocrinology and Metabolism Research Center, Shiraz University of Medical Sciences, Shiraz, Iran; 2 Molecular Pathology and Cytogenetics Division, Department of Pathology, Shiraz University of Medical Sciences, Shiraz, Iran; 3 Endocrinology and Metabolism Research Center, Nemazee Hospital, Shiraz University of Medical Sciences, Postal Box: 71345-1414, Shiraz, Iran

**Keywords:** Ocular, Melanoma, Papillary thyroid cancer, Case report

## Abstract

**Background:**

Melanoma develops in the cells that produce melanin; ocular melanoma accounts for 3–4% of all malignant melanomas. Thyroid tumors are the most common endocrine neoplasms, with more than 95% of cases arising from follicular cell origin. Previous studies have reported associations between malignant melanoma and a wide variety of malignancies.

**Case presentation:**

We report a 54-year-old Iranian woman who was diagnosed with ocular melanoma based on a mushroom-shaped filling defect with homogeneous echo pattern arising from the anterior third of the temporal side of the globe detected on ocular sonography during routine ophthalmological examination. She underwent right globe enucleation and implant replacement. During tumor surveillance, fluorodeoxyglucose positron emission tomography/computed tomography scan showed low-grade metabolically active tumoral involvement in the anterolateral aspect of the right lobe of thyroid. The patient subsequently underwent thyroidectomy and submandibular lymphadenectomy. Pathologic report demonstrated micropapillary carcinoma (9 × 8 mm^2^), tall cell variant without lymphovascular or perineural invasion in the base of lymphocytic thyroiditis.

**Conclusion:**

This case illustrates the importance of precise active surveillance in case of papillary carcinoma of thyroid or malignant melanoma to avoid missing other associated pathologies and emphasizes the simultaneous treatment of two tumors.

## Background

Melanoma is a type of cancer that develops from melanin-producing cells, which can arise in the eyes. Ocular melanoma accounts for 3–4% of all malignant melanomas [1]. There are two major subtypes of ocular melanoma: uveal melanoma, which arise from the iris, choroid, and ciliary body, and conjunctival melanoma, whose origin is the conjunctiva [2]. This malignant tumor can potentially disperse through the body and cause distant metastasis, most often in the liver [3].

Thyroid tumors are the most common endocrine neoplasms. More than 95% of them develop from follicular cell origin. In patients with widespread malignancy, bloodborne metastases to the thyroid are reported in 0.5–24.2% in autopsy studies, but these metastatic lesions are rarely detectable clinically [4].

There are some reports of associations between malignant melanoma and other malignancies, including second primary melanoma, nonmelanoma skin cancer, central nervous system tumors, Hodgkin’s lymphoma, non-Hodgkin’s lymphoma, leukemia, breast, and ovarian carcinoma [5]. Ozgun *et al.* reported a case with malignant melanoma and papillary thyroid carcinoma diagnosed concurrently and treated simultaneously [6].

The present study reports another case of this rare co-occurrence of malignant melanoma and papillary thyroid carcinoma, which were treated simultaneously.

## Case presentation

A 54-year-old Iranian woman, married, resident in Shiraz, Fars Province in the south of Iran, referred to the ophthalmologist for routine eye examination. Her past medical history included only type II diabetes mellitus treated with 1000 mg metformin and 50 mg sitagliptin daily. The patient’s social history was negative for cigarette smoking or alcohol use. Her family history for malignancy was negative.

Due to suspicious lesion on dilated eye examination by ophthalmologist, sonography was requested. Sonographer reported a mushroom-shaped filling defect with homogeneous echo pattern arising from the anterior third of the temporal side of the globe wall, suggestive of melanoma; this 10 × 9.8 mm^2^ lesion could arise from the ciliary body and the choroid.

**Fig. 1:**
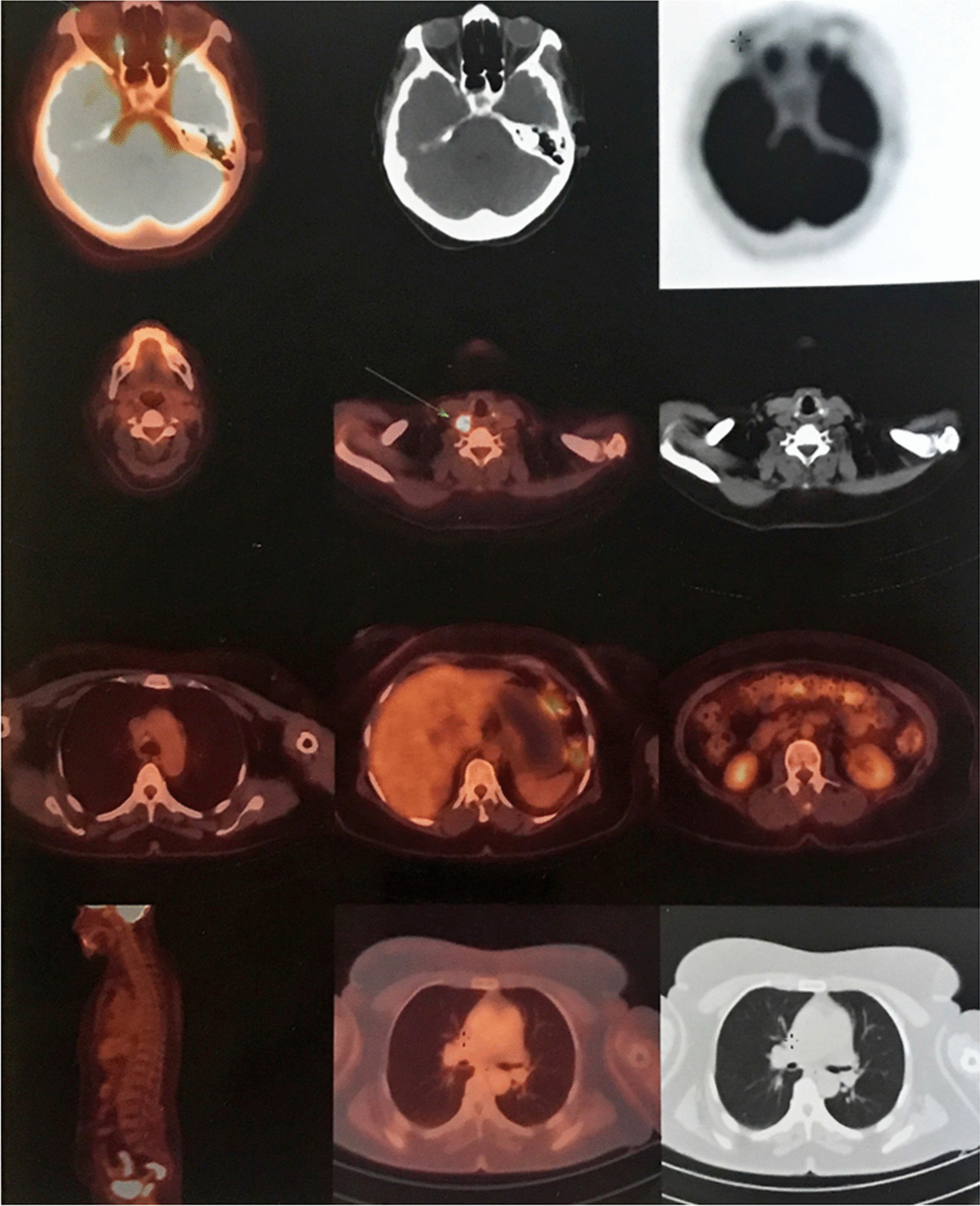
FDG-PET/CT scan: low-grade metabolically active tumoral involvement in the anterolateral aspect of the right lobe of thyroid as well as hypermetabolic lymphadenopathy at cervical zone VI

In tumor surveillance, thoracic and abdominal CT scan with IV contrast were normal. FDG-PET/CT scan showed low-grade metabolically active tumoral involvement in the anterolateral aspect of the right lobe of thyroid as well as hypermetabolic lymphadenopathy at cervical zone VI (Fig. 1).

**Fig. 2:**
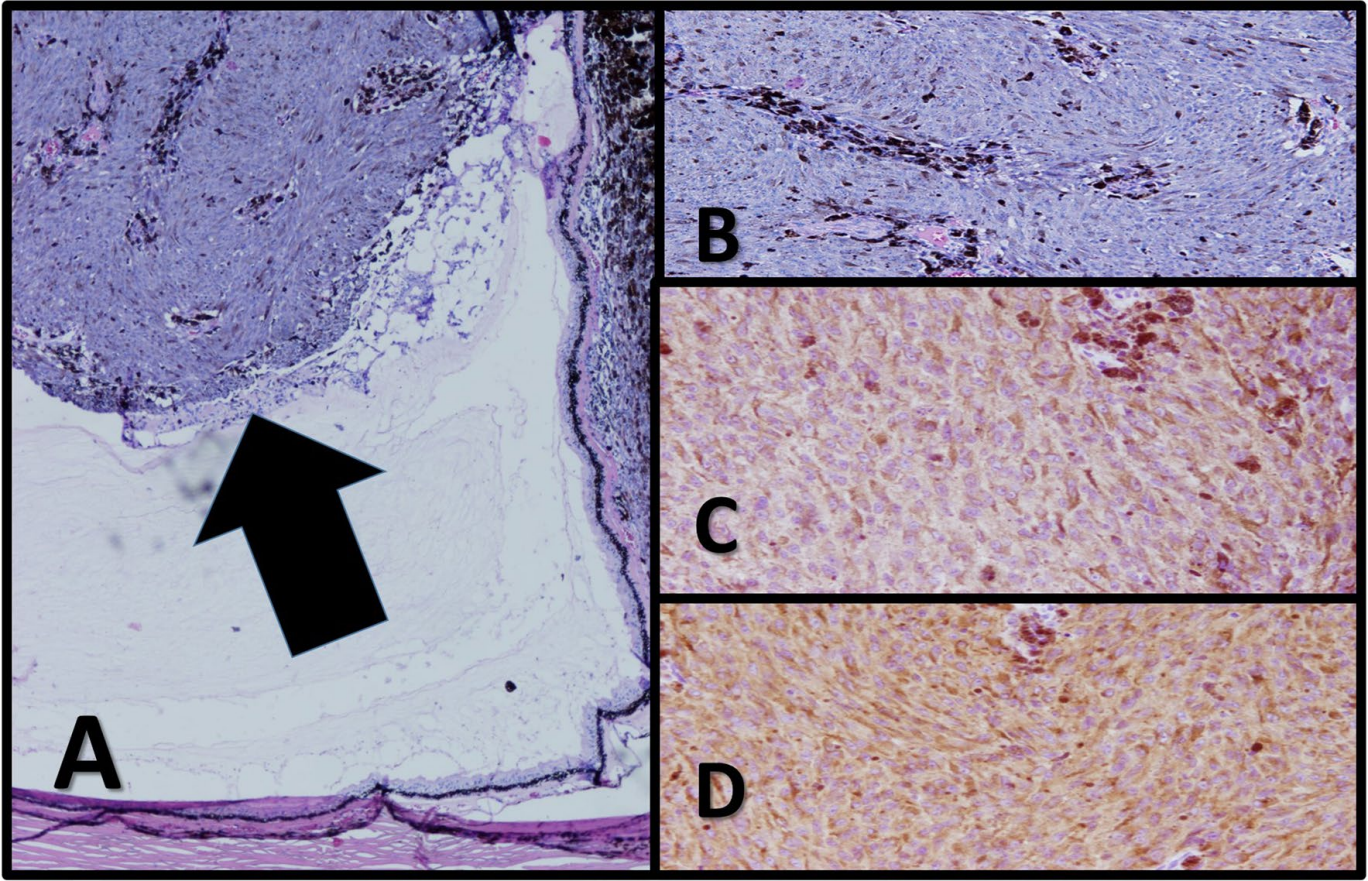
Histopathology of the uveal tumor. **A**, **B** Histopathological sections show a mushroom-shaped tumoral process (arrow) arising from retina composed of highly pleomorphic plump spindle cells with some dense melanin pigments in the background. Individual cells show high degree of nuclear atypia with prominent nucleoli. Tumor infiltrating lymphocyte and macrophage is less than 5%. Vessels show loop pattern (×40, ×100 H&E). **C**, **D** Immunohistochemical staining of the tumor shows diffuse, strong immunoreactivity for Melan-A and HMB-45 (×100, ×100)

Right globe enucleation and implant replacement was performed for the patient; pathologic report revealed 9 × 10 mm^2^ malignant melanoma in choroid anterotemporal area with 0–1 mitotic area per 10 high-power fields (HPF) and involvement of anterior chamber and focal retina. Histologic type was mixed epitheloid (25%) and spindle cell melanoma (75%). Tumor cells were positive for HMB45 and Melan A. According to the AJCC 8th edition, tumor wasT3aN0M0, G1, at least stage IIB (Fig. 2).

High-resolution ultrasonography of the neck soft tissue revealed normal thyroid gland without any sign of solid or cystic lesion, and a suspicious enlarged significant hypoechoic lymph node measuring about 12 × 12 mm^2^ in the right jugular chain at zone II. Sonography-guided fine-needle aspiration biopsy of this lesion revealed some clusters of malignant looking cells with high N/C ratio, hyperchromasia, and a few nuclear inclusions, suggestive of malignancy.

At this time, right-side parotidectomy and fascial nerve trunk exploration and right-side modified neck dissection was performed.

Pathologic report showed parotid gland without specific pathologic change, and seven lymph node labeled as level II and III; and four lymph node labeled as level IV, which were all reactive lymph nodes without evidence of malignancy.

One month later, high-resolution ultrasonography of the thyroid and neck showed an oval-shaped hypoechoic structure measuring 13 mm in right para tracheal area attached or invading right border of thyroid capsule with central echogenic hilum and central internal hypervascularity, which seems to be metastatic or suspicious lymph node.

**Fig. 3:**
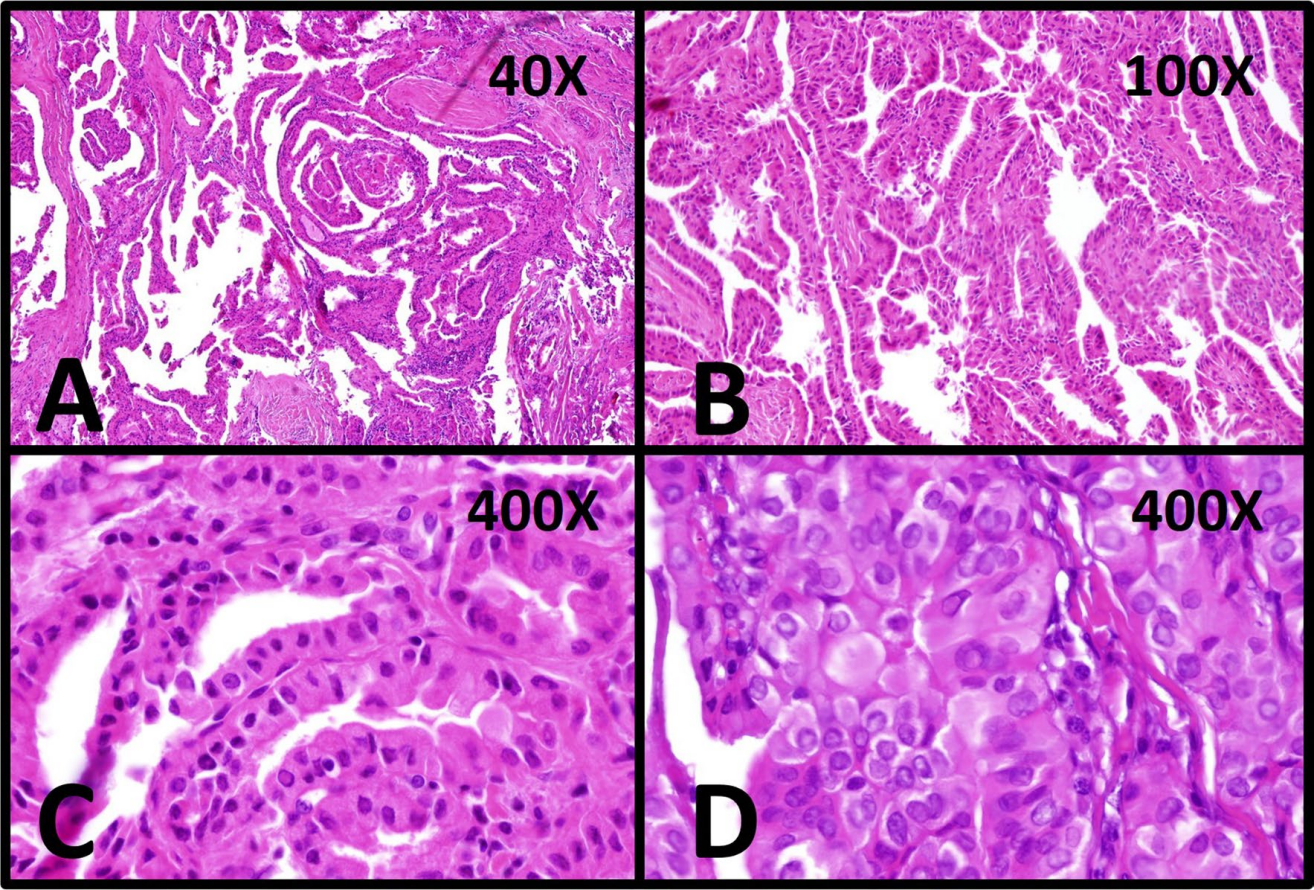
Histopathology features of the thyroid tumor: **A**, **B** Histopathological sections show papillary architecture with minimal stromal lymphocytic infiltration and fibrous bandings (H&E stained, ×40, ×100). **C**, **D** Each papillae lined with columnar epithelium with round to oval nuclei with relatively clear chromatins and nuclear pseudoinclusions (arrows) sufficient for diagnosis of conventional-type papillary thyroid carcinoma (H&E stained, ×400, ×400)

The patient subsequently underwent thyroidectomy and submandibular lymphadenectomy. Pathologic report demonstrated micropapillary carcinoma (9 × 8 mm^2^), tall cell variant without lymphovascular or perineural invasion in the base of lymphocytic thyroiditis. One tissue labeled as right cervical lymph node revealed soft tissue with chronic inflammation and foreign body giant cell reaction (Fig. 3). Postoperative ultrasonography of the neck did not show any evidence of lymphadenopathy.

**Fig. 4:**
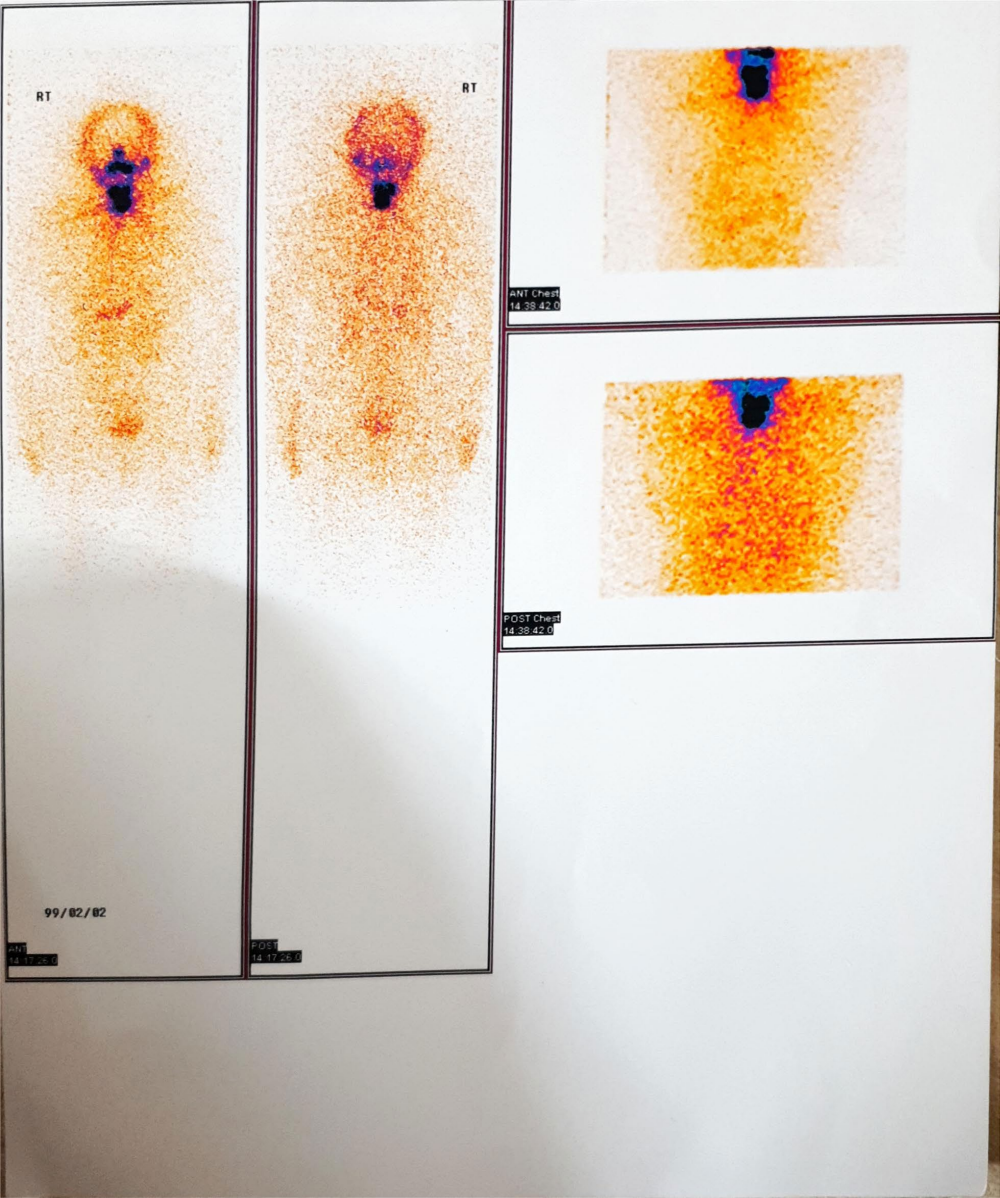
Whole-body I-131 scan: evidence of thyroid remnant and possibility of cervical lymph node involvement without evidence of distant metastases

Seventy-five days after thyroidectomy, 125-mCi I-131 was administered to the patient, who was off levothyroxine for 4 weeks. Whole-body scan after 7 days showed evidence of thyroid remnant and possibility of cervical lymph node involvement without evidence of distant metastases (Fig. 4). At time of iodine administration, she had TSH > 100 mIU/ml, thyroglobulin < 0.04 ng/ml, and negative antithyroglobulin antibody. Eight months later, whole-body scan 2 days after oral administration of 5-mCi I-131 did not show any evidence of abnormal radioiodine-avid lesion throughout the body. At time of iodine administration, she had TSH > 100 mIU/ml, thyroglobulin < 0.2 ng/ml, and negative antithyroglobulin antibody. At this time, the patient is in good health state and takes levothyroxine with daily dose of 125 µg.

## Discussion

Malignant melanoma is reported to be associated with second primary malignancies such as prostate, soft tissue, salivary gland, and musculoskeletal cancers [7]. Although thyroid carcinoma is not a common secondary malignancy in melanoma, it is shown that its risk increases 2.17-fold among patients with malignant melanoma [8]. On the other hand, papillary carcinoma of thyroid increases the risk of melanoma by 1.8-fold [9].

Various probable mechanisms have been described for an association between papillary thyroid carcinoma and melanoma. Thyroid stimulating hormone (TSH), which is increased in thyroid failure, can stimulate melanocyte growth and division via TSH receptors on their surface [10]. Recent studies have revealed that coincidence of thyroid papillary carcinoma and malignant melanoma is associated with *BRAF* gene mutation. *BRAF* gene is associated with aggressive subtypes. In a review on the role of *BRAF* in pathogenesis of papillary thyroid carcinoma and cutaneous melanoma, Mitchell *et al.* concluded that the BRAF protein acts as a catalyst for epithelial–mesenchymal transition in both malignancies [11]. Detection of this mutation is important since BRAF inhibitor medications such as vemurafenib and dabrafenib can be useful in their treatment [12]. Another mutation that is reported to increase the risk of uveal melanoma and thyroid carcinoma is BRCA-1 associated protein (BAP1). This mutation is found more among families with familial cancer syndrome [13].

Although papillary thyroid carcinoma and malignant melanoma can occur with a time interval similar to our case, they have presented simultaneously in some cases where they were treated together [6, 14]. Previous studies reported cases of concomitant cutaneous malignant melanoma and papillary thyroid carcinoma. However, in our case, the origin of the malignant melanoma was the ciliary body and the choroid tissue.

It is worth mentioning that malignant melanoma can show similar pathologic features as papillary carcinoma of thyroid, with a pseudopapillary appearance, which is formed by cuffs of tumor cells surrounding stromal vessels [15, 16]. There are several studies reporting misdiagnosis and consequently mismanagement of such cases. It is thus important to distinguish these two pathologies, especially in case of amelanotic malignant melanoma with papillary features using immunohistochemical tests. One of the rare differential diagnoses of thyroid mass following malignant melanoma is metastasis, which was reported before in a 63-year-old man [17]. Therefore, it is crucial to identify the nature of the mass carefully.

## Conclusion

There is some evidence in favor of associations between malignant melanoma and a wide variety of malignancies, including papillary thyroid carcinoma. Our patient demonstrated these two cancers concomitantly. This case illustrates the importance of precise active surveillance in case of papillary carcinoma of thyroid or malignant melanoma to avoid missing other associated pathologies. In addition, it should be considered that the treatment of either tumor should not be delayed and the two tumors can be treated simultaneously.

## Data Availability

The datasets used and analyzed during the present study are available from the corresponding author on reasonable request.
